# Rietveld refinement of the mixed boracite Fe_1.59_Zn_1.41_B_7_O_13_Br

**DOI:** 10.1107/S1600536809044407

**Published:** 2009-10-31

**Authors:** Sandra Ulloa-Godínez, Ivonne Rosales, Lauro Bucio, Mario H. Farías, Jorge Campa-Molina

**Affiliations:** a Centro Universitario de Ciencias Exactas e Ingenierá, Universidad de Guadalajara, Laboratorio de Materiales Avanzados del Departamento de Electrónica, Av. Revolución No. 1500, Módulo O, Planta baja, Zona Olímpica, CP 44840, Guadalajara, Jalisco, Mexico; b Facultad de Ingeniería, Universidad Autóoma de Baja California, Blvd. Benito Juarez s/n col. Insurgentes, Mexicali, Baja California, Mexico; c Instituto de Física, Universidad Nacional Autónoma de México, AP 20-364, 01000 México DF, Mexico; d Centro de Nanociencias y Nanotecnología, Universidad Nacional, Autónoma de México, Apdo. Postal 356, CP 22800, Ensenada, Baja California, Mexico

## Abstract

The structural characterization of the new iron–zinc hepta­borate bromide with composition Fe_1.59_Zn_1.41_B_7_O_13_Br, prepared by chemical transport is reported. A rigid-body model with constrained generalized coordinates was defined in order to hold the positions of the B atoms at reasonable inter­atomic distances that typically would reach unacceptable values because of the weak scattering power of boron. There are three independent sites for the B atoms of which two are tetra­hedrally coordinated. The bond-valence sum around the third B atom, located on a threefold rotation axis, was calculated considering two cases of coordination of boron with oxygens: trigonal-planar and tetrahedral. The contribution of the fourth O atom to the bond-valence sum was found to be only 0.06 v.u., indicating the presence of a very weak bond in the right position to have a distorted tetra­hedral coordination in favour of the trigonal-planar coordination for the third B atom. X-ray fluorescence (XRF) was used to determinate the Fe/Zn ratio.

## Related literature

The method of preparation was based on Schmid (1965 ▶). For related structures, see: Mao *et al.* (1991 ▶); Dowty & Clark (1972 ▶, 1973 ▶); Mendoza-Alvarez *et al.* (1985 ▶); Schindler & Hawthorne (1998 ▶); Knorr *et al.* (2007 ▶). For properties and potential applications of boracites, see: Campa-Molina *et al.* (1994 ▶, 2002 ▶); Dana (1951 ▶); Mathews *et al.* (1997 ▶); Smart & Moore (1992 ▶). For bond-valence parameters for oxides, see: Brese & O’Keeffe (1991 ▶).

## Experimental

### 

#### Crystal data

Fe_1.59_Zn_1.41_B_7_O_13_Br
*M* *_r_* = 544.65
Trigonal, 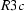

*a* = 8.6081 (1) Å
*c* = 21.0703 (3) Å
*V* = 1352.12 (3) Å^3^
*Z* = 6
Cu *K*α radiation
*T* = 300 K
Specimen shape: irregular
20 × 20 × 0.2 mm
Specimen prepared at 1173 K
Particle morphology: irregular, pale pink

#### Data collection

Bruker D8 Advance diffractometer
Specimen mounting: packed powder sample container
Specimen mounted in reflection mode
Scan method: step
2θ_min_ = 8.1, 2θ_max_ = 110.0°
Increment in 2θ = 0.02°

#### Refinement

*R*_p_ = 0.018
*R*_wp_ = 0.025
*R*_exp_ = 0.014
*R*_B_ = 0.06
*S* = 1.89
Profile function: pseudo-Voigt modified by Thompson *et al.* (1987 ▶)
397 reflections
18 parameters

### 

Data collection: *DIFFRAC/AT* (Siemens, 1993 ▶); cell refinement: *FULLPROF* (Rodríguez-Carvajal, 2006 ▶; Rodriguez & Rodriguez-Carvajal, 1997 ▶, a strongly modified version of that described by Wiles & Young, 1981); data reduction: *FULLPROF*; method used to solve structure: coordinates were taken from an isotypic compound (Mao *et al.*, 1991 ▶); program(s) used to refine structure: *FULLPROF*; software used to prepare material for publication: *DIAMOND*.

## Supplementary Material

Crystal structure: contains datablocks global, I. DOI: 10.1107/S1600536809044407/br2119sup1.cif

Rietveld powder data: contains datablocks I. DOI: 10.1107/S1600536809044407/br2119Isup2.rtv

Additional supplementary materials:  crystallographic information; 3D view; checkCIF report

**Fig. 4.**
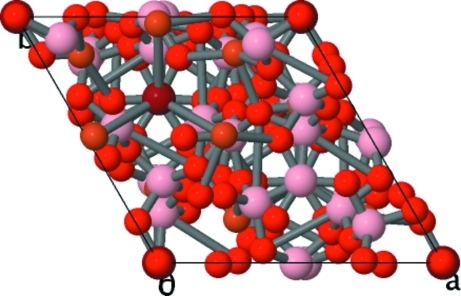
Crystal structure of Fe1.59Zn1.41B7O13Br mixed boracite

Enhanced figure: interactive version of Fig. 4

## Figures and Tables

**Table:** 

Zn—Br	2.680 (3)
Zn—Br^i^	3.412 (1)
Zn—O2^ii^	2.130 (4)
Zn—O3^iii^	2.081 (7)
Zn—O4^iv^	2.035 (4)
Zn—O5^v^	2.012 (7)
B1—O2^vi^	1.506 (14)
B1—O3^vii^	1.48 (2)
B1—O4^vii^	1.451 (13)
B1—O5^vi^	1.49 (3)
B2—O1	1.566 (3)
B2—O3^viii^	1.452 (8)
B2—O4	1.463 (18)
B2—O5	1.453 (17)
B3—O2	1.397 (14)
B3—O1	2.38 (3)

**Table:** 

O2—B3—O2^vii^	119.9 (9)

Symmetry codes: (i) 
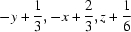
; (ii) 
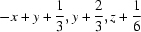
; (iii) 
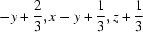
; (iv) 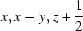; (v) 
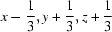
; (vi) 
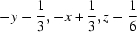
; (vii) 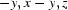; (viii) 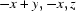.

**Fig. 1.**
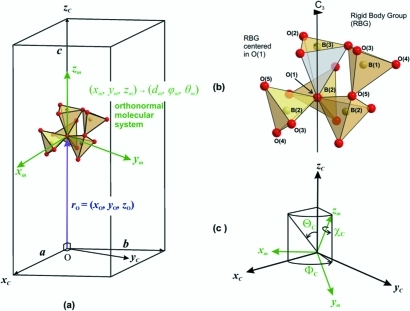
(a) Rigid body group (RBG) and the coordinate systems defined for performing the movements of the RBG as a whole, are represented in the unit cell of the Fe1.59Zn1.41B7O13Br mixed boracite. (b) Atoms belonging to the RBG: some are labelled and the remainig are related by symmetry (there is a symmetry axis C3 crossing vertically the RBG). (c) Orthogonal coordinate system to perform the rotations of the RBG and orthonormal molecular system.

**Fig. 2.**
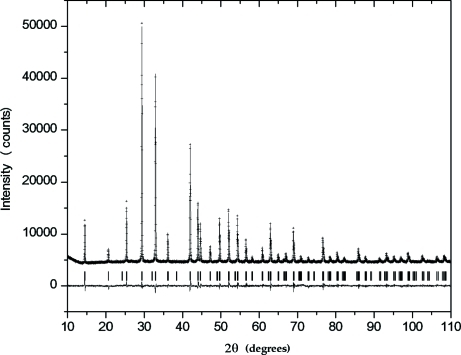
Observed (crosses), calculated (solid line) and difference (solid line at the bottom) from the final Rietveld refinement of the X-ray powder diffraction data of Fe1.59Zn1.41B7O13Br mixed boracite at room temperature. Vertical marks correspond to the positions allowed for the Bragg reflections.

**Fig. 3.**
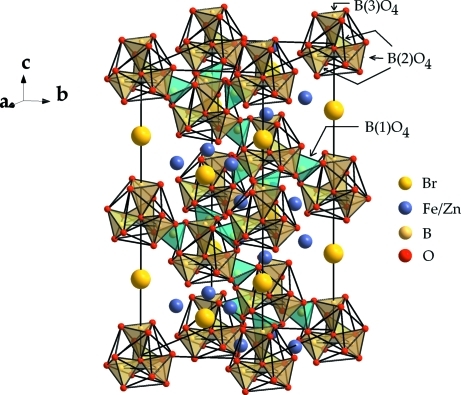
Structural representation of Fe1.59Zn1.41B7O13Br mixed boracite.

## supplementary crystallographic information

### Comment

The new iron-zinc heptaborate bromine Fe_1.59_Zn_1.41_B_7_O_13_Br, belongs to the family of boracites with general formula Me_3_B_7_O_13_*X*, where *Me* could be a divalent ion and *X* a halogen. Boracites have attracted the attention of researchers since Häuy, who observed pyroelectricity in the mineral boracite Mg_3_B_7_O_13_Cl (Dana, 1951). Unusual physical properties can be cited for a given cations located in the crystalographic sites for Me and X. Depending on this, potential applications such as an optic stopper (Smart & Moore, 1992); ferroelectric non volatile memory (Mathews, *et al.*, 1997); and infrared (IR) detection (Campa -Molina *et al.*, 1994, 2002) have been reported and in some sense, can be modulated by the presence of some specific types of cations. The aim of this research was to synthesize a new boracite with Zn and Fe in the crystalographic sites for *Me* in the general formula, in order to stablish in first instance, its structural and crystal chemistry properties. They are needed for the understanding of its physical properties. The representation of the crystal structure of the Fe_1.59_Zn_1.41_B_7_O_13_Br mixed boracite appear in figure 3. Bond valence calculations were made using the recommended bond-valence parameters for oxides published by Brese & O'Keeffe (1991) and considering those coordination polyhedra whose bond valence calculations were based on distances and angles that were allowed to refine (this was partially true in some cases).  Bond valence sum around Br is found to be 0.82 and 1.10, for BrZn_6_ and BrFe_6_ distorted octahedra respectively. The resulting average value is then 0.97 if a composition of 53% Fe and 47% Zn is considered for this site, which is almost equal to the expected value of 1 for Br. Around the Fe/Zn site, four O atoms and Br are coordinated. The bond valence sums that result here are 1.97 and 1.76 when the site is only occupyied by Fe and Zn respectively. The average value for 53% Fe and 47% Zn is then 1.87 with good proximity to the expected value of 2. For the B(3) atom, the contribution of the fourth oxygen atom O(1) to the bond valence sum obtained for the B(3)O3 triangle of 2.8 is increased to 2.86 (i.e. only 0.06 v. u. indicating the presence of a very weak bond in the right position for have a distorted tetrahedral coordination around the planar triangle coordination for the third boron atom B(3). This fact is also a feature for the reported boracites Fe_3_B_7_O_13_Cl (ICSD 60504, Mendoza-Alvarez *et al.*, 1985), and Zn_3_B_7_O_13_Cl (ICSD 55444, Mao *et al.*, 1991).

### Experimental

Single crystals of Fe_1.59_Zn_1.41_B_7_O_13_Br were grown by a chemical vapour transport technique, commonly called the three-crucibles method, reported by Schmid (1965). Growth takes place in a closed quartz ampoule. Chemical transport reactions were carried out by heating the ampoule at about 1173 K in a resistance-heated vertical furnace, with gradients of 850 K (above) and 650 K (below), over a period of 72 h. The reactants were placed in the following order: 1.7 g of B_2_O_3_ (which was obtained by dehydrating H_3_BO_3_) was placed in the first crucible; 0.5 g of each one of both metal oxides (ZnO and FeO) in the second crucible; and 0.8 g of each one of both divalent metal halides (FeCl_2_ and ZnCl_2_) in the third crucible. Crystals of Fe_1.59_Zn_1.41_B_7_O_13_Br as large as 2 mm in size were commonly obtained. X-ray Fluorescence (XRF) spectroscopy was used to estimate the Fe/Zn ratio. A small crystallite was irradiated using the "SANDRA" system developed at Instituto de Fisica, UNAM, equipped with a 75 W Mo X-ray tube (50 kV, 1.5 mA, XTF5011 model from Oxford Instruments) and AmpTeK Si-Pin detector. The system was calibrated using reference standard materials from *NIST* (SRM 2711). The average percent atomic content with standard uncertainty for each element in the sample were 53 (4) % for iron, and 47 (4) % for zinc, and give a Fe:Zn ratio of 1.13. Then the stichiometric formula is Fe_1.59 (12)_Zn_1.41 (12)_B_7_O_13_Br.

### Refinement

The characterization of powdered Fe_1.59_Zn_1.41_B_7_O_13_Br mixed boracite by conventional X-ray powder diffraction data indicated the presence of a well crystallized phase showing reflections that matched with the isostructural phase trembathite, Mg_1.56_Fe_1.44_Mn_0.02_B_7_O_13_Cl (PDF 01–089-6198) reported by Schindler & Hawthorne (1998). The starting structural parameters to perform a Rietveld refinement of the Fe_1.59_Zn_1.41_B_7_O_13_Br boracite were taken from the isostructural data reported for Zn_3_B_7_O_13_Cl (ICSD 55444) by Mao *et al.* (1991). The following parameters were refined: zero point, scale factor, background parameters, unit cell dimensions, half-width, pseudo-Voigt and asymmetry parameters for the peak shape; position and thermal isotropic factors. For the case of boron, the thermal isotropic factors were fixed to 0.24 Å^2^, which is a reasonable value for the boron atom and for obtaining a good refinement. The occupation factors for Fe and Zn atoms sharing the same position were fixed to the values of 0.53 and 0.47 respectively, obtained by a quantitative chemical analysis from X-ray fluorescence (XRF) spectroscopy. Due to the very low scattering power of boron atoms to the X-rays, one rigid body group (RBG) containing the boron atoms was defined as ilustrated in figure 1. This RBG has its centre in O(1) atom. Then, eight atoms define the complete RGB (including the centre) and are labelled as B(1), B(2), B(3), O(1), O(2), O(3), O(4) and O(5). Each atom has their spherical internal coordinates (d*_m_*, φ*_m_*, θ*_m_*) fixed according to the rigid character of the RBG formed by these eight atoms. The parameters χ_c_, Θ_c_, Φ_c_, *x*_o_, *y*_o_, *z*_o_, that were refined in a first step are represented in fig. 1 b, c and were limited by the symmetry allowed movements for the RBG as a whole. At the end of this step, B(1)O_4_, B(2)O_4_ tetrahedra, and B(3)O_3_ triangle kept their interatomic angles and distances. In a second and final step of refinement the spherical internal coordinates for B(3) and O(2) were refined in such a way to allow to bring the B(3)O_3_ triangle closer to the O(1) atom. The RBG subroutine has been included in the program FULLPROF (Rodriguez & Rodriguez-Carvajal, 1997). The use of the RBG reduced significantly the number of positional parameters in the Rietveld refinement. The results of the refinement are shown in figure 2.

### Crystal data

**Table:**

Fe_1.59_Zn_1.41_B_7_O_13_Br	*F*(000) = 1546.0
*M**_r_* = 544.65	*D*_x_ = 4.013 Mg m^−^^3^
Trigonal, *R*3*c*	Cu *K*α radiation, λ = 1.54175 Å
Hall symbol: R 3 -2"c	*T* = 300 K
*a* = 8.6081 (1) Å	Particle morphology: irregular
*c* = 21.0703 (3) Å	pale pink
*V* = 1352.12 (3) Å^3^	irregular, 20 × 20 mm
*Z* = 6	Specimen preparation: Prepared at 1173 K

### Data collection

**Table:**

Bruker D8 Advance diffractometer	Data collection mode: reflection
Radiation source: sealed X-ray tube, Cu Kα	Scan method: step
graphite	2θ_min_ = 8.12°, 2θ_max_ = 110.01°, 2θ_step_ = 0.02°
Specimen mounting: packed powder sample container	

### Refinement

**Table:**

Least-squares matrix: full with fixed elements per cycle	5240 data points
*R*_p_ = 0.018	Profile function: pseudo-Voigt modified by Thompson *et al.* (1987)
*R*_wp_ = 0.025	18 parameters
*R*_exp_ = 0.014	Weighting scheme based on measured s.u.'s
*R*_Bragg_ = 0.06	(Δ/σ)_max_ = 0.02
*R*(*F*^2^) = 0.06	Background function: linear interpolation between a set of 72 background points with refinable heights
χ^2^ = 3.572	

### Fractional atomic coordinates and isotropic or equivalent isotropic displacement parameters (Å^2^)

**Table:**

	*x*	*y*	*z*	*U*_iso_*/*U*_eq_	Occ. (<1)
Br	0.00000	0.00000	0.26670	0.0084 (9)	
Zn	0.1488 (8)	0.3037 (3)	0.3347 (2)	0.0113 (8)	0.47000
Fe	0.1488 (8)	0.3037 (3)	0.3347 (2)	0.0113 (8)	0.53000
B1	−0.184 (1)	0.1519 (1)	−0.080 (1)	0.00304	
B2	0.1130 (2)	−0.0902 (1)	−0.0259 (1)	0.00304	
B3	0.00000	0.00000	0.105 (1)	0.00304	
O1	0.00000	0.00000	−0.008 (1)	0.0025 (9)	
O2	0.010 (1)	−0.157 (2)	0.107 (1)	0.0025 (9)	
O3	0.282 (1)	0.274 (1)	−0.032 (1)	0.0025 (9)	
O4	0.206 (1)	−0.006 (1)	−0.085 (1)	0.0025 (9)	
O5	0.242 (1)	−0.059 (1)	0.024 (1)	0.0025 (9)	

### Geometric parameters (Å, °)

**Table:**

Zn—Br	2.680 (3)	B1—O4^vii^	1.451 (13)
Zn—Br^i^	3.412 (1)	B1—O5^vi^	1.49 (3)
Zn—O2^ii^	2.130 (4)	B2—O1	1.566 (3)
Zn—O3^iii^	2.081 (7)	B2—O3^viii^	1.452 (8)
Zn—O4^iv^	2.035 (4)	B2—O4	1.463 (18)
Zn—O5^v^	2.012 (7)	B2—O5	1.453 (17)
B1—O2^vi^	1.506 (14)	B3—O2	1.397 (14)
B1—O3^vii^	1.48 (2)	B3—O1	2.38 (3)
			
Zn—Br—Zn^vii^	94.1 (2)	O2—B3—O2^vii^	119.9 (9)

Symmetry codes:  (i) −*y*+1/3, −*x*+2/3, *z*+1/6; (ii) −*x*+*y*+1/3, *y*+2/3, *z*+1/6; (iii) −*y*+2/3, *x*−*y*+1/3, *z*+1/3; (iv) *x*, *x*−*y*, *z*+1/2; (v) *x*−1/3, *y*+1/3, *z*+1/3; (vi) −*y*−1/3, −*x*+1/3, *z*−1/6; (vii) −*y*, *x*−*y*, *z*; (viii) −*x*+*y*, −*x*, *z*.
